# Impact of Hand Fractures on Return to Skate, Performance, Time on Ice, and Physicality in the National Hockey League

**DOI:** 10.7759/cureus.104907

**Published:** 2026-03-09

**Authors:** Chloe Heiting, Priya Duvvuri, George Economou, Sruthi Katakam, Camille Pinpin, Carolina Herrera, Brian Li, Kate W Nellans

**Affiliations:** 1 Orthopedic Surgery, Donald and Barbara Zucker School of Medicine at Hofstra/Northwell, Hempstead, USA; 2 Orthopedic Surgery, Northwell Health, New Hyde Park, USA; 3 Orthopedic Surgery, Rutgers New Jersey Medical School, Newark, USA

**Keywords:** finger fracture, hand fracture, ice hockey, ice hockey injury, return to skate, sports medicine, sports performance, wrist fracture

## Abstract

Introduction

Hand fractures in ice hockey players have the potential to profoundly impact performance, but the associations between specific fracture characteristics and performance have yet to be quantified. The objective of this study is to assess the rates of hand fractures in the National Hockey League (NHL) over the past 25 years and determine the impact of fracture site and laterality on return to skate (RTS), games missed, performance, and time on ice (TOI) after injury.

Methods

Carpal, metacarpal, and phalangeal fractures sustained by active players were retrospectively collected from the 2000-2001 through 2024-2025 seasons of the NHL using the public database Pro Sports Transactions. Date of injury, RTS, games missed, injury laterality, performance metrics (Corsi, Fenwick, and PDO), TOI, and hits were collected and verified using player game logs and team schedules. RTS, games missed, change in performance, TOI, and hits were compared between fracture site and laterality.

Results

There were 170 fractures reported over 25 seasons (24 carpal, 58 metacarpal, 88 phalanx), with significantly more fractures occurring in the non-dominant shooting hand. The average RTS was 34.1 days, with players missing an average of 12.1 games. Phalanx fractures had a significantly faster RTS than carpal or metacarpal fractures. Overall, Corsi and Fenwick performance metrics significantly decreased after hand fractures, whereas hits and short- and long-term TOI were not significantly affected.

Conclusion

Hand fractures can result in significant declines in NHL player performance after RTS. Fractures to the non-dominant hand may be particularly detrimental to players, resulting in a slower RTS than fractures to the dominant hand. These insights highlight potential focus areas for prevention and rehabilitation protocols for ice hockey players with hand fractures.

## Introduction

Professional ice hockey is a fast-paced contact sport in which the National Hockey League (NHL) comprises 32 teams across the United States (US) and Canada, each maintaining an active roster of 23 players [1,2]. As hockey has grown in popularity, so too has the skill level, speed, and physicality of its athletes. Skating speeds regularly reach 20 miles per hour, and pucks are shot at upwards of 100 miles per hour [3]. Although the protective equipment worn by players has similarly advanced, overall injury incidence has been reported to range from 14.2-15.6 per 1000 athlete-game exposures [4,5].

Hand injuries are common in ice hockey, accounting for a substantial proportion of overall injuries occurring at elite athletic levels and comprising 14.1% of all ice hockey injuries requiring emergency evaluation [3,6,7]. Incidence of forearm, hand, and wrist injuries ranges from 0.51-0.8 per 1000 athlete-game exposures and is especially detrimental due to the crucial role of the upper extremity in ice hockey [5,8]. While both hands are important in stick grip and torque, they play different roles in stick and puck handling. Specifically, the lower hand on the stick generates power and stability while the upper hand offers stick control.

Despite the potentially detrimental implications of these injuries, return to skate (RTS) and performance after hand fractures have only recently been explored [9]. NHL players suffering hand fractures often miss games and are driven to rapidly RTS, perhaps due to personal and team financial incentives [10]. Prior work has demonstrated that NHL players with metacarpal and phalangeal fractures actually miss fewer games on average compared to National Basketball Association (NBA) players [9]. Despite this short recovery period, they have been found to successfully return to pre-injury performance within one year of the initial injury. However, the long-term implications of these injuries have not been described. Further, the impact of the injury laterality presents a relevant and largely unexplored factor.

This study aims to describe the prevalence of hand fractures within the NHL and compare the effect of fractures to the carpal, metacarpal, and phalanx on RTS, games missed, time on ice (TOI), performance, and physicality. The prevalence and impact of fractures to the dominant versus non-dominant shooting hand will also be explored to better inform the player, trainer, and clinician on the management of hand fractures in NHL players. We hypothesized that fractures to the dominant versus non-dominant hands would demonstrate differing impacts on RTS and player performance, and predicted minimal changes in TOI and physicality after RTS regardless of the fracture laterality and site.

## Materials and methods

Injuries sustained by NHL players from the 2000-2001 through 2024-2025 NHL seasons were evaluated. Injury reports for carpal, metacarpal, and phalanx fractures were retrospectively analyzed from the publicly available Pro Sports Transactions database [11], which has previously been used in the analysis of injuries in the NHL [12]. The date of injury and/or movement to the injured reserve was extracted from the database, and injuries were each independently verified by three reviewers using official NHL or team press releases and media reports. Player demographics, including date of birth, position at time of injury, and shooting hand, were collected from the team roster.

Additional player data, including number of regular season games missed, date of RTS, average TOI, average hits per season, and three player performance metrics (Corsi, Fenwick, and PDO) were then collected from the player and team game logs on Hockey-Reference and externally verified as described above [13].

Change in average TOI after fracture was calculated as short- or long-term. The short-term was calculated as the difference between the average TOI of the five games preceding and following the game during which the fracture occurred. Long-term was calculated as the difference between the average TOI of the seasons preceding and following the season of injury. Player performance was assessed beginning in the 2008-2009 season, utilizing the Corsi percentage, Fenwick percentage, and PDO calculated for all ice situations (i.e., even-strength and player-advantage play). These metrics have previously been utilized to evaluate the performance of NHL players after injury [14], and were created and recorded beginning in 2007 to better describe the scoring chances produced by a player [15]. Further, they may be used to evaluate both forwards and defensemen, whereas traditional performance metrics such as goals, assists, or points per game reflect inherent differences in positional roles and ice time. Corsi percentage represents the percentage of shots taken by a player’s team out of all shots taken by both teams while a player is on the ice. The Fenwick percentage is more nuanced, only including shots that were not blocked. PDO has been described as the “puck luck” of ice hockey and is the sum of the team’s save percentage and shooting percentage. Player physicality was assessed by the average number of hits recorded for the season preceding and following the fracture. To account for differences in player TOI, hits were adjusted and presented as average hits per average TOI. Injuries that resulted in failure of the player to RTS at least five games before the end of the regular season were classified as season-ending injuries (SEIs) and analyzed separately.

A total of 193 carpal, metacarpal, and phalanx fractures were identified. Three players were excluded from the final cohort due to fractures occurring outside of the NHL season, and 20 were excluded due to missing data (i.e., unable to verify injury). A total of 85 players were injured in the final season of their career or otherwise did not have data for the season following their injury, and were excluded from the TOI analysis. A total of 73 players were either injured after performance metrics (Corsi percentage, Fenwick percentage, PDO) and hits were first recorded in 2007, were injured in the final season of their career, or were missing this data and were therefore excluded from the performance and hits analyses. Seven goaltenders with hand fractures were also excluded from these analyses due to their low prevalence in the cohort and the lack of these data points owing to the nature of their position.

Descriptive statistics were performed to characterize injury prevalence, RTS, and games missed. Chi-squared test of independence was used to compare the proportion of dominant versus non-dominant hand fractures. One-way ANOVA was used to compare RTS, games missed, performance metrics, and hits between fracture sites and injury laterality. Independent t-tests were utilized to compare performance metrics and hits between dominant and non-dominant hands. Paired t-tests were performed to compare performance metrics and hits between the seasons preceding and following injury. Statistics were performed using IBM SPSS Statistics Version 27.0 (IBM Corp., Armonk, NY). The significance level was p < 0.05.

## Results

RTS and games missed

**Figure 1:**
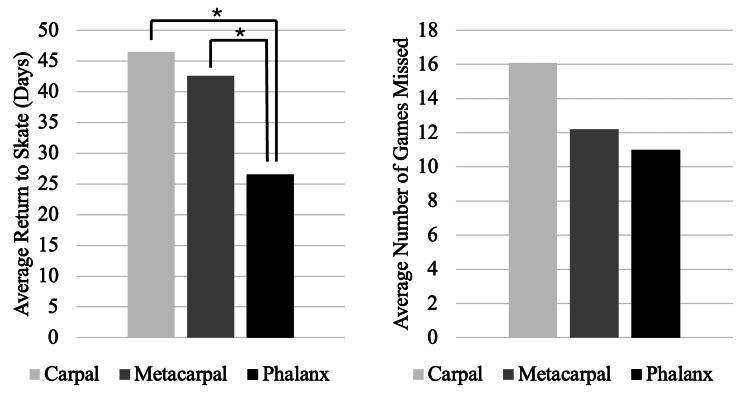
Average return to skate and games missed, stratified by fracture site. Asterisk indicates significant difference (p < 0.05).

There were 170 hand fractures identified from the 2000-2001 through 2024-2025 NHL seasons, 30 (17.6%) of which were SEIs (Table 1). The most prevalent fracture was to the phalanx (51.8%), followed by the metacarpal (34.1%), then the carpal (14.1%). Stratification by position revealed that forwards (left wing, center, right wing) sustained more injuries than both defense and goalies. The average RTS after a hand fracture was 34.1 days, and players missed an average of 12.1 games. Despite being the most prevalent fracture site, phalanx fractures resulted in the fewest average games missed (11.0 games) and a significantly faster RTS (26.5 days) than carpal and metacarpal fractures (p = 0.002 and p < 0.001, respectively) (Figure 1).

**Table 1:** Injury demographics by injury type. Data presented as n (%) unless otherwise specified. ^a^Data presented as mean (SD). RTS, return to skate; SEI, season-ending injury

Fracture Type	Injury (n)			Position at Time of Injury					Average RTS (Days)^a^	Average Games Missed^a^
	All	Non-SEI	SEIs	Left Wing	Center	Right Wing	Defense	Goalie		
Carpal	24	19 (79.2%)	5 (20.8%)	5 (20.8%)	7 (29.2%)	3 (12.5%)	9 (37.5%)	0 (0.0%)	46.5 (21.7)	16.1 (9.38)
Metacarpal	58	44 (75.9%)	14 (24.1%)	14 (24.1%)	12 (20.7%)	4 (6.9%)	27 (46.6%)	1 (1.7%)	42.6 (29.4)	12.2 (6.46)
Phalanx	88	77 (87.5%)	11 (12.5%)	19 (21.6%)	19 (21.6%)	14 (15.9%)	30 (34.1%)	6 (6.8%)	26.5 (12.3)	11.0 (8.02)
Total	170	140 (82.4%)	30 (17.6%)	38 (22.4%)	38 (22.4%)	21 (12.4%)	66 (38.8%)	7 (4.1%)	34.1 (21.9)	12.1 (7.88)

Stratification of hand fractures by shooting hand revealed that there was a greater incidence of fractures in non-dominant than dominant hands, χ²(1, N = 94) = 4.26, p = 0.039, and φ = 0.213 (Table 2). SEIs were also more common in the non-dominant hand compared to the dominant hand, χ²(1, N = 81) = 3.57, p = 0.059, and φ = 0.210. The greatest proportion of non-dominant fractures was to the phalanges (47.9%), whereas metacarpal fractures were most prevalent amongst dominant hand injuries (44.4%). Shooting hand laterality also demonstrated differing impacts on RTS and games missed after fracture. Fractures to the dominant hand resulted in a faster average RTS than injuries to the non-dominant hand, although this was not significant (p = 0.212). The average number of games missed was similar between dominant and non-dominant hands for each fracture site.

**Table 2:** Injury demographics stratified by injury type and laterality. Data presented as n (%) unless otherwise specified. ^a^Data presented as mean (SD). ^b^Comparison between dominant and non-dominant injuries. RTS, return to skate; SEI, season-ending injury

Fracture Type	Hand Injury Laterality	Injury (n)			Average RTS (Days)^a^	p-value^b^	Average Games Missed^a^	p-value^b^
		All	Non-SEIs	SEIs				
Carpal	Dominant	7	5 (71.4%)	2 (28.6%)	57.8 (23.5)	0.357	20.4 (9.1)	0.678
	Non-dominant	6	6 (100%)	0 (0.0%)	45.5 (18.6)		18.3 (3.4)	
Metacarpal	Dominant	16	13 (81.2%)	3 (18.8%)	33.4 (8.9)	0.133	11.5 (5.1)	0.275
	Non-dominant	25	19 (76.0%)	6 (24.0%)	51.1 (40.5)		13.6 (6.4)	
Phalanx	Dominant	14	14 (100%)	0 (0.0%)	23.7 (12.9)	0.188	9.9 (6.1)	0.729
	Non-dominant	26	24 (92.3%)	2 (7.7%)	29.4 (12.7)		10.7 (6.5)	
Total	Dominant	37	32 (86.5%)	5 (13.5%)	32.7 (17.6)	0.212	12.5 (7.3)	0.881
	Non-dominant	57	49 (86.0%)	8 (14.0%)	39.8 (28.8)		12.8 (7.0)	

Short- and long-term TOI

Though not statistically significant, fractures to the hand resulted in an average loss of 0.44 minutes of average long-term TOI (p = 0.150), whereas short-term TOI was relatively unchanged (Table 3). There was a decrease in average short- and long-term TOI after non-dominant hand injuries, regardless of fracture site, although this was also not significant. Comparison of fracture sites and fracture to the dominant versus non-dominant hand did not demonstrate a significant difference in short- or long-term change in TOI (Table 3).

**Table 3:** Average change in short- and long-term TOI (minutes) by fracture type and injury laterality. Data presented as mean (SD). ^a^Hand injury laterality was not reported for all injuries. ^b^Comparison between dominant and non-dominant injuries. TOI, time on ice

Fracture Type	Hand Injury Laterality^a^	n	Average Short-Term Change in TOI (Minutes)	p-value^b^	Average Long-Term Change in TOI (Minutes)	p-value^b^
Carpal	All Injuries	11	-0.36 (1.80)	0.527	-0.008 (1.70)	0.989
	Dominant	4	1.21 (1.37)	0.174	1.28 (1.80)	0.159
	Non-dominant	3	-0.34 (1.15)		-0.73 (1.19)	
Metacarpal	All Injuries	26	0.63 (3.15)	0.317	-0.76 (3.31)	0.253
	Dominant	9	1.00 (2.07)	0.238	-0.32 (2.54)	0.326
	Non-dominant	12	-0.42 (2.99)		-1.45 (2.51)	
Phalanx	All Injuries	48	-0.22 (2.38)	0.525	-0.36 (2.71)	0.355
	Dominant	11	-0.37 (2.63)	0.801	0.14 (2.13)	0.682
	Non-dominant	14	-0.62 (2.36)		-0.37 (3.58)	
Total	All Injuries	85	0.02 (2.58)	0.936	-0.44 (2.79)	0.150
	Dominant	24	0.41 (2.30)	0.173	0.16 (2.23)	0.176
	Non-dominant	29	-0.51 (2.49)		-1.46 (2.76)	

Performance, hits, and games played after RTS 

There was a significant decrease in player performance as measured by Corsi and Fenwick after hand fractures (p = 0.004 and p = 0.004, respectively), where Corsi decreased by 1.90, and Fenwick decreased by 1.98 (Table 4). Stratification by injury site revealed that there was a significant decrease in Corsi and Fenwick for players with carpal fractures (p = 0.050 and p = 0.041, respectively) and Corsi for players with metacarpal fractures (p = 0.045). There was no significant difference in PDO for any fracture site. Performance metrics did not significantly differ between fracture sites or injury laterality. 

**Table 4:** Change in performance metrics (Corsi percentage, Fenwick percentage, and PDO) for all hand fractures and fractures stratified by injury site. Change calculated using seasons preceding and following the season of injury. Data presented as mean (SD).

Fracture Type	n	Performance Metric					
		Change in Corsi %	p-value	Change in Fenwick %	p-value	Change in PDO	p-value
Carpal	12	-3.48 (5.73)	0.050	-3.90 (5.82)	0.041	-1.33 (5.26)	0.402
Metacarpal	40	-1.78 (5.43)	0.022	-1.47 (5.67)	0.050	1.62 (9.27)	0.139
Phalanx	45	-1.58 (7.10)	0.071	-1.92 (7.51)	0.046	0.01 (7.51)	0.987
Total	97	-1.90 (6.26)	0.004	-1.98 (6.59)	0.004	0.51 (6.95)	0.474

The average number of TOI-adjusted hits after hand fractures slightly increased from 0.075 to 0.077 hits per minute of ice time, although there was no significant change even after stratification by fracture site (Table 5). However, NHL players performed a significantly greater number of TOI-adjusted hits after fractures to the non-dominant metacarpal (p < 0.001) and phalanx (p < 0.001). There was no significant change in games played after a hand fracture (Table 5). 

**Table 5:** Change in TOI-adjusted hits and games played for all hand fractures and fractures stratified by injury site. Change calculated using seasons preceding and following the season of injury. Data presented as mean (SD). TOI, time on ice

Fracture Type	n	Change in Hits (Hits/Minute TOI)	p-value	Changes in Games Played	p-value
Carpal	12	0.010 (0.06)	0.593	9.43 (30.36)	0.233
Metacarpal	40	-0.004 (0.06)	0.676	1.07 (26.14)	0.794
Phalanx	45	0.006 (0.05)	0.416	3.86 (30.46)	0.323
Total	97	0.002 (0.05)	0.662	3.65 (28.9)	0.171

## Discussion

Our study demonstrated that hand fractures are a common and detrimental injury for NHL players, with differing implications for RTS and post-injury performance depending on the fracture site and injury laterality. Although performance as measured by Corsi and Fenwick scores significantly decreased during the season following a hand fracture, there was no significant change in short- and long-term TOI, regardless of stratification by fracture site or injury laterality. We also found that player physicality, assessed by TOI-adjusted hits, did not decline after fracture and even significantly increased after fractures to the non-dominant metacarpal and phalanx. Our study provides a novel exploration of the effects of hand fractures on player TOI and physicality and builds upon the limited literature examining the effect of hand fractures on RTS and performance.

Phalanx fractures were the most prevalent in our cohort and yielded a significantly faster RTS than carpal and metacarpal fractures. While the quick RTS may reflect a less important role of the phalanx in stick grip and maneuvering, it may also be facilitated by the incorporation of a splint or cast within a hockey glove or the modification of equipment to minimize use of the injured phalanx [16,17]. Numerous articles describe the importance of early controlled motion to limit stiffness and promote healing after fracture [18,19], and these adjustments may allow for the balance between splint/cast immobilization and early motion as the player resumes play. However, this rapid RTS may have long-term repercussions and prove detrimental to NHL player performance. Although not significant, Corsi and Fenwick performance scores and short- and long-term average TOI decreased after phalanx fractures. The potential ramifications of phalanx fractures may be underestimated, perhaps due to the prevalence of these injuries or the ability of players to quickly RTS.

Metacarpal and carpal fractures resulted in a lengthier time to RTS compared with phalanx fractures, but also demonstrated significant decreases in at least one of the examined performance metrics. Literature describing metacarpal fracture management in ice hockey suggests cast immobilization for four to six weeks, or until the player can grip the stick, and this recovery period may be doubled for carpal fractures [20]. This timeline aligns with our finding that the average RTS after a carpal or metacarpal fracture exceeds 40 days and may explain the significant decline in player performance, given the longer period without training. This detrimental effect on performance, despite a longer recovery phase, may reflect greater severity of injuries to these sites, poor healing environments in bones like the scaphoid, and reduced ability to integrate splints/casts with the player’s equipment.

Our finding that player performance decreased after hand fractures was in contrast with the work of Gotlin and colleagues, who found that NHL players returned to pre-injury performance within one year of hand, wrist, or forearm fracture [9]. While this may be, in part, due to the differing metrics used to evaluate performance, our study also examined a decade of more recent NHL seasons. We hypothesize that our incongruent findings reflect a changing epidemiology of hand fractures in NHL players, possibly due to advancements in protective equipment or improvements in therapeutic protocols and interventions. There were, however, several consistent findings between our study and that of Gotlin et al. The mean number of games missed after fracture was similar between our studies, and neither cohort demonstrated a significant change in games played after fracture.

Our exploration of the effect of hand dominance provides nuanced insight into fracture management and suggests that there may be different mechanisms and consequences of fractures to the dominant versus non-dominant shooting hand. There were significantly more fractures to the non-dominant than the dominant hand in our cohort, suggesting a greater propensity of injury to the non-dominant hand in ice hockey. Interestingly, the prevalence of dominant versus non-dominant fractures also appears to differ in other stick- or racket-wielding sports. For example, hamate fractures were found to be more common in the dominant hand of tennis players and in the non-dominant hand of baseball players [21,22].

Lastly, we observed a longer RTS, a greater number of games missed, and a decrease in TOI after non-dominant compared to dominant shooting hand fractures, though these trends were not significant. These findings may reflect the differing roles of the dominant and non-dominant hands in ice hockey. The lower hand on the stick, which dictates shooting hand dominance, generates power and stability, while the upper hand controls the stick. Further, the upper non-dominant shooting hand is constantly gripping the stick, whereas the lower dominant hand may be released when a player is reaching with their stick or swinging their arms to increase skating speed. Injuries to these respective hands may therefore have different ramifications on stick and puck handling. Taken together, our findings highlight potential differences in therapeutic protocols and player perceptions surrounding hand injuries. It is possible that players, trainers, and clinicians are more intentional and aggressive in their management of dominant hand injuries due to the dominant hand’s clear role in stick-wielding, thereby minimizing the deleterious effect on player RTS and games missed.

Our study is not without limitations. The injury data was obtained from a public database and verified from public sources, including official NHL or team press releases and media reports. Excluding injuries that lacked accurate and consistent information also contributed to a smaller data set. For example, the NHL does not require that detailed data be provided for player injuries, and many injuries were described only as “upper body injury” and excluded from our analysis. We also utilized three separate metrics (Corsi percentage, Fenwick percentage, and PDO) to assess player performance. Although these metrics have previously been utilized to assess post-injury performance [14], they were only reported after the 2007 season and favor offensive performance due to their inclusion of a team’s “shots for” into the calculation. Additionally, TOI may be affected by confounding factors, such as player performance or team strategy and dynamics. Lastly, there were substantially fewer injuries in the early years of our cohort due to a lack of media reporting and database documentation. There were also fewer injuries in the seasons played during the coronavirus pandemic, but we included these seasons to comprehensively report the hand injury epidemiology in the NHL.

## Conclusions

Hand fractures can be detrimental injuries for NHL players, at times resulting in a significant decrease in player performance after RTS. The impact of the injury on player RTS, performance, and physicality differs based on the fracture site and laterality. Notably, non-dominant shooting hand fractures result in a significantly longer RTS than fractures to the dominant hand. Our study suggests that these injury characteristics should be considered by players, coaching staff, and team physicians and trainers to inform and improve rehabilitation and RTS protocols. Future studies can compare mechanisms of injury between the non-dominant and dominant shooting hands.
